# Replication Study in a Japanese Population of Six Susceptibility Loci for Type 2 Diabetes Originally Identified by a Transethnic Meta-Analysis of Genome-Wide Association Studies

**DOI:** 10.1371/journal.pone.0154093

**Published:** 2016-04-26

**Authors:** Ren Matsuba, Minako Imamura, Yasushi Tanaka, Minoru Iwata, Hiroshi Hirose, Kohei Kaku, Hiroshi Maegawa, Hirotaka Watada, Kazuyuki Tobe, Atsunori Kashiwagi, Ryuzo Kawamori, Shiro Maeda

**Affiliations:** 1 Laboratory for Endocrinology, Metabolism and Kidney Diseases, RIKEN Center for Integrative Medical Sciences, Yokohama, Japan; 2 Department of Internal Medicine, Division of Metabolism and Endocrinology, St. Marianna University School of Medicine, Kawasaki, Japan; 3 First Department of Internal Medicine, University of Toyama, Toyama, Japan; 4 Health Administration Center, University of Toyama, Toyama, Japan; 5 Health Center, Keio University School of Medicine, Tokyo, Japan; 6 Department of Internal Medicine, Kawasaki Medical School, Kurashiki, Japan; 7 Department of Medicine, Shiga University of Medical Science, Otsu, Japan; 8 Department of Metabolism and Endocrinology, Juntendo University Graduate School of Medicine, Tokyo, Japan; 9 Sportology Center, Juntendo University Graduate School of Medicine, Tokyo, Japan; 10 Department of Advanced Genomic and Laboratory Medicine, Graduate School of Medicine, University of the Ryukyus, Nishihara, Japan; 11 Division of Clinical Laboratory and Blood Transfusion, University of the Ryukyus Hospital, Nishihara, Japan; Wake Forest School of Medicine, UNITED STATES

## Abstract

**Aim:**

We performed a replication study in a Japanese population to evaluate the association between type 2 diabetes and six susceptibility loci (*TMEM154*, *SSR1*, *FAF1*, *POU5F1*, *ARL15*, and *MPHOSPH9*) originally identified by a transethnic meta-analysis of genome-wide association studies (GWAS) in 2014.

**Methods:**

We genotyped 7,620 Japanese participants (5,817 type 2 diabetes patients and 1,803 controls) for each of the single nucleotide polymorphisms (SNPs) using a multiplex polymerase chain reaction invader assay. The association of each SNP locus with the disease was evaluated using logistic regression analysis.

**Results:**

Of the six SNPs examined in this study, four (rs6813195 near *TMEM154*, rs17106184 in *FAF1*, rs3130501 in *POU5F1* and rs4275659 near *MPHOSPH9*) had the same direction of effect as in the original reports, but two (rs9505118 in *SSR1* and rs702634 in *ARL15*) had the opposite direction of effect. Among these loci, rs3130501 and rs4275659 were nominally associated with type 2 diabetes (rs3130501; p = 0.017, odds ratio [OR] = 1.113, 95% confidence interval [CI] 1.019–1.215, rs4275659; p = 0.012, OR = 1.127, 95% CI 1.026–1.238, adjusted for sex, age and body mass index), but we did not observe a significant association with type 2 diabetes for any of the six evaluated SNP loci in our Japanese population.

**Conclusions:**

Our results indicate that effects of the six SNP loci identified in the transethnic GWAS meta-analysis are not major among the Japanese, although SNPs in *POU5F1* and *MPHOSPH9* loci may have some effect on susceptibility to type 2 diabetes in this population.

## Introduction

Diabetes mellitus affects nearly 400 million individuals worldwide, and its prevalence is progressively increasing in both developing and developed countries, including Japan [1]. Although the pathogenesis of type 2 diabetes, which is the most common form of the disease, has not yet been fully elucidated, the combination of insulin resistance in peripheral tissues and impairment of insulin secretion from pancreatic islets is considered a principal cause. Cummulative evidence has suggested that genetic factors play an important role in the pathogenesis of type 2 diabetes, and extensive efforts are being made to identify genetic loci conferring susceptibility to type 2 diabetes [2].

In 2007, five independent groups performed genome-wide association studies (GWAS) for type 2 diabetes using populations of European descent; they identified *TCF7L2*, which had previously been discovered via positional cloning in 2006 [3], as well as eight other genetic loci that affect susceptibility to type 2 diabetes, namely *SLC30A8*, *HHEX*, *CDKN2A/B*, *CDKAL1*, *IGF2BP2*, *KCNJ11*, *PPARG*, and *FTO* [4–8]. Subsequently, these study groups have been attempting to identify additional susceptibility loci with smaller effect sizes by increasing sample sizes for European GWAS, and nearly 50 susceptibility loci to type 2 diabetes have been confirmed through these studies [9–12]. Most of these loci have been consistent in their effects across non-European populations including the Japanese [13–20], but associations of some loci with type 2 diabetes were not replicated, suggesting differences of genetic background among different ethnic groups.

In 2008, two Japanese GWAS simultaneously identified the *KCNQ1* as a strong susceptibility locus for type 2 diabetes [21, 22]. Although large-scaled European GWAS meta-analyses had not detected it, the *KCNQ1* locus was shown to be a common susceptibility locus to type 2 diabetes in several ethnic groups including European populations [21, 22], indicating the importance of performing GWAS in various ethnic groups. Larger-scaled Japanese GWAS have been conducted, and six additional susceptibility loci to type 2 diabetes have been identified: *UBE2E2*, *C2CD4A*-*C2CD4B*, *ANK1*, *SLC16A13*, *LEP*-*MIR129* and *GPSM1* [23–25]. The former three loci were significantly associated with European type 2 diabetes, but the latter three were not [25], highlighting the possibility of genetic heterogeneity between Japanese and European populations with regard to type 2 diabetes.

In 2014, a transethnic GWAS meta-analysis which combined GWAS data from multiple ethnic groups, including European, East Asian, South Asian and Mexican/Mexican American, identified seven novel loci for type 2 diabetes [26]: rs6813195 near *TMEM154*, rs9505118 in *SSR1*, rs17106184 in *FAF1*, rs3130501 in *POU5F1*, rs6808574 near *LPP*, rs702634 in *ARL15*, and rs4275659 near *MPHOSPH9*. Since this study addressed problems of sample size and genetic heterogeneity by performing a large-scale meta-analysis over a variety of ethnic groups, these seven SNP loci are likely to be common susceptibility loci for type 2 diabetes across all these groups (European, East Asian, South Asian and Mexican/Mexican American). However, the 18,820 East Asian individuals, 6,954 type 2 diabetes cases and 11,866 controls, used in the transethnic GWAS meta-analysis consisted of several different subpopulations, including Han Chinese (n = 10,717), Korean (n = 3,985), Filipino (n = 1,783), and Japanese (n = 2,335), and it has been suggested that genetic heterogeneity may also exist to some degree among subpopulations of East Asian descent [27]. Therefore, the contribution of the seven loci for susceptibility to type 2 diabetes needs to be evaluated independently in each subpopulation.

In this study, we evaluated the effects of the seven loci identified by the transethnic GWAS meta-analysis in an independent Japanese population by examining the association of these loci with type 2 diabetes.

## Materials and Methods

### Participants and DNA Preparation

We enrolled 5,817 type 2 diabetes patients who regularly visited the outpatient clinics of the Shiga University of Medical Science, Kawasaki Medical School, St. Marianna University, Juntendo University, and the University of Toyama or who were registered with BioBank Japan [23]. Diabetes mellitus was diagnosed according to the World Health Organization (WHO) criteria [28], and type 2 diabetes was clinically defined as gradual, adult-onset diabetes. Patients who tested positive for antibodies to glutamic acid decarboxylase, or who were diagnosed with a monogenic form of the disease, such as mitochondrial disease or maturity-onset diabetes of the young, were excluded from the present study. We also recruited 1,803 controls who underwent annual health checks at Keio University, St. Marianna University, or Toyama University Hospital. Genomic DNA was extracted from peripheral blood using a standard phenol-chloroform procedure.

### Ethics Statements

All participants agreed to the protocol of this study and provided written informed consent. This protocol was approved by the ethics committees of the RIKEN Yokohama Institutes and each of the participating institutes: Shiga University of Medical Science, Kawasaki Medical School, St. Marianna University, Juntendo University, the University of Toyama, and Keio University.

### SNP Genotyping

We selected seven autosomal single nucleotide polymorphisms (SNPs) identified by transethnic GWAS meta-analysis in 2014, including rs6813195 near *TMEM154*, rs9505118 in *SSR1*, rs17106184 in *FAF1*, rs3130501 in *POU5F1*, rs6808574 near *LPP*, rs702634 in *ARL15*, and rs4275659 near *MPHOSPH9* [26]. Out of these seven SNPs, rs6808574 near *LPP* was excluded from this analysis because it was monoallelic in the Japanese population (rs6808574-C 100% in Hapmap phase 3 *JPT*: http://hapmap.ncbi.nlm.nih.gov/ and 1000 genomes project phase 3 *JPT*: http://www.1000genomes.org/).

Genotyping was performed using a multiplex-polymerase chain reaction (PCR) invader assay as reported previously [29, 30]. The genotyping success rates for all of the SNPs were over 98.0% (S1 Table). The genotype concordance rates in the 64 duplicated samples were 100%.

### Statistical Analysis

Statistical analyses were performed using methods described in our previous study [30]. We applied Hardy-Weinberg equilibrium (HWE) tests according to the protocol described by Nielsen et al. [31]. Differences in the genotype distribution of each SNP between cases and controls were evaluated using logistic regression analyses with and without adjustments for age, sex, and body mass index (BMI). The association of each SNP with quantitative traits, including fasting plasma glucose (FPG), insulin (IRI), the homeostasis model assessment of beta-cell function (HOMA-β), and HOMA of insulin resistance (HOMA-IR) [32, 33], was evaluated using multiple linear regression analysis. Because the values of these traits in the experimental Japanese population displayed skewed distribution, we used log-transformed values for analyses. Genotypes of each SNP were scored using an additive model (0, 1, and 2 for the homozygous of non-effect allele, heterozygous, and homozygous of effect allele, respectively). Statistical analyses were performed using StatView software (SAS Institute, Cary, NC, USA). Significance was determined by Bonferroni’s method for correcting multiple testing errors; p < 0.0083 (0.05 divided by 6) was therefore considered statistically significant. We estimated the statistical power of our study at different expected odds ratios based on the sample size of our study (n = 7,620) and allele frequencies obtained from a public database (1000 genomes project, phase 3 *JPT* http://browser.1000genomes.org/ind3x.html). The sample size in this study (n = 7,620) meets the 80% statistical power (α = 0.05/6 = 0.0083) for the six SNP loci assuming each of their effect sizes (odds ratios) was 1.2, and for the four SNP loci (rs6813195, rs9505118, rs3130501 and rs4275659) assuming their odds ratio was 1.1. However, if the effect sizes of individual SNP loci had been assumed to be the same as those in the original study [26], the estimated statistical power of this study would have been between 23% and 58% (α = 0.05, S2 Table).

## Results

Clinical characteristics of the participants are shown in Table 1. The genotype distributions of all SNPs were in accordance with Hardy-Weinberg equilibrium proportions (S3 Table).

**Table 1:** Clinical characteristics of participants.

	Sample size (case/control)	Type 2 diabetes	Controls	*p* value
n		5,817	1,803	
Sex (M:F)		3,616: 2,201	864: 939	< 0.0001^b^
Age (year)^a^	5,817/1,803	63.3 ± 11.3	49.7 ± 17.0	< 0.0001^b^
BMI (kg/m^2^)^a^	5,817/1,803	24.2 ± 4.0	22.4 ± 3.2	< 0.0001^b^
HbA1c (%)^a^	4,624/570	8.1 ± 2.6	5.3 ± 0.3	< 0.0001^c^
FPG (mmol/L) ^a^	1,938/1,144	8.3 ± 2.9	5.3 ± 0.6	< 0.0001^c^
Duration of diabetes ^a^	3,779/-	13.5 ± 9.7	-	

^a^ Data are mean ± standard deviation.

^b^ Student’s unpaired t-test

^c^ Mann-Whitney U test

BMI: body mass index, HbA1c: Glycated hemoglobin, FPG: fasting plasma glucose

Of the six SNPs exmamied, four (rs6813195 near *TMEM154*, rs17106184 in *FAF1*, rs3130501 in *POU5F1*, and rs4275659 near *MPHOSPH9*) had the same direction of effect (odds ratio > 1.0, adjusted for sex, age, and BMI; Table 2, S4 Table) as previously reported, but two (rs9505118 in *SSR1* and rs702634 in *ARL15*) had the opposite direction of effect (odds ratio < 1.0; p = 0.2344, binomial test; Table 2, S4 Table). Among them, rs3130501 and rs4275659 were nominally associated with type 2 diabetes (rs3130501, p = 0.017, odds ratio [OR] = 1.113, 95% confidence interval [CI] 1.019–1.215; and rs4275659, p = 0.012, OR = 1.127, 95% CI 1.026–1.238; both adjusted for sex, age, and body mass index (BMI); Table 2). The remaining 4 SNPs were not associated with type 2 diabetes in this study (p ≥ 0.05, adjusted for sex, age and BMI; Table 2).

**Table 2:** Association of 6 SNP loci with type 2 diabetes in a Japanese population.

SNP	Nearby gene	Risk Allele^a^	RAF	Unadjusted		Adjusted^b^	
				*p* value	OR(95%CI)	*p* value	OR (95%CI)
rs6813195	*TMEM154*	C	0.47	0.062	1.075 (0.996–1.159)	0.096	1.077 (0.987–1.174)
rs9505118	*SSR1*	A	0.56	0.935	0.997 (0.924–1.075)	0.513	0.971 (0.890–1.060)
rs17106184	*FAF1*	G	0.90	0.589	1.037 (0.909–1.183)	0.616	1.040 (0.893–1.210)
rs3130501	*POU5F1*	G	0.57	0.012	1.103 (1.021–1.191)	0.017	1.113 (1.019–1.215)
rs702634	*ARL15*	A	0.82	0.962	1.002 (0.908–1.107)	0.848	0.989 (0.883–1.108)
rs4275659	*MPHOSPH9*	C	0.67	0.010	1.114 (1.027–1.209)	0.012	1.127 (1.026–1.238)
GRS^c^^,^ ^d^				2.4×10^−3^	1.056 (1.020–1.094)	0.014	1.052 (1.010–1.095)

The results of logistic regression analysis are shown.

^a^ Risk allele reported in the original trans-ethnic GWAS.

^b^ Adjusted for age, sex and BMI.

^c^ The genetic risk score (GRS) was calculated according to the number of risk alleles by counting the 6 trans-ethnic genome-wide association study derived SNPs

^d^ Individuals who had complete genotype data for 6 SNPs (n = 7,293) were used for the analyses

We further examined the association of the six SNPs with the quantitative glycemic traits, HOMA-IR, HOMA-β, IRI, and FPG using control participants. The risk allele for type 2 diabetes of rs17106184 in *FAF1* was nominally associated with an increase in the HOMA-IR value (0.0083 ≤ p < 0.05 adjusted for sex, age and BMI; Table 3). The remaining five SNP loci were not associated with any glycemic traits in this study (p ≥ 0.05, adjusted for sex, age and BMI; Table 3).

**Table 3:** Association of 6 SNP loci with quantitative traits related to glucose metabolism in control individuals.

SNP	Nearby gene	Risk Allele^a^	HOMA-IR^b^ (n = 802)		HOMA-β^b^ (n = 802)		FPG^b^ (n = 1,148)		IRI^b^ (n = 867)	
			Effect (SE)	*p* value	Effect (SE)	*p* value	Effect (SE)	*p* value	Effect (SE)	*p* value
rs6813195	*TMEM154*	C	-0.032 (0.024)	0.175	-0.018 (0.025)	0.466	-0.002 (0.004)	0.705	-0.032 (0.022)	0.152
rs9505118	*SSR1*	A	-0.020 (0.024)	0.405	-0.001 (0.025)	0.982	-0.004 (0.004)	0.312	-0.010 (0.023)	0.673
rs17106184	*FAF1*	G	0.083 (0.041)	0.043	0.044 (0.043)	0.308	0.004 (0.008)	0.578	0.051 (0.039)	0.187
rs3130501	*POU5F1*	G	3.8E-4 (0.024)	0.987	-0.019 (0.025)	0.450	0.004 (0.004)	0.406	-0.003 (0.022)	0.906
rs702634	*ARL15*	A	-0.007 (0.032)	0.827	-0.016 (0.033)	0.639	-0.006 (0.006)	0.283	-0.006 (0.030)	0.843
rs4275659	*MPHOSPH9*	C	0.033 (0.025)	0.192	0.031 (0.027)	0.244	-0.008 (0.005)	0.081	0.031 (0.024)	0.206

The results of linear regression analysis with adjustment for age, sex and BMI are presented.

^a^ Risk allele reported in the original trans-ethnic GWAS

^b^ log-transformed values were applied for the analyses

## Discussion

We examined the association of six SNP loci, rs6813195 near *TMEM154*, rs9505118 in *SSR1*, rs17106184 in *FAF1*, rs3130501 in *POU5F1*, rs702634 in *ARL15*, and rs4275659 near *MPHOSPH9*, identified by transethnic GWAS meta-analysis with susceptibility to type 2 diabetes in a Japanese population. While rs3130501 in *POU5F1* and rs4275659 near *MPHOSPH9* were nominally associated with type 2 diabetes, none of the six SNPs was significantly associated with the disease in this population (Table 2).

The transethnic GWAS meta-analysis from the original report had the largest sample size of 110,455 participants (26,490 cases and 83,965 controls), including European (n = 69,033), East Asian (n = 18,820), South Asian (n = 20,019) and Mexican/Mexican American (n = 2,583) populations, and the identified seven loci were found to be common susceptibility loci for type 2 diabetes across all these ethnic groups [26]. However, only 2,335 Japanese individuals were included in that meta-analysis. In addition, it has been suggested that East Asian subpopulations differ to some degree with regards to the genetic compornent of predisposition to type 2 diabetes [27]; therefore, the association of the seven loci with type 2 diabetes needs to be evaluated in a larger, solely Japanese population sample. Indeed, risk allele fequencies for rs6808574 near *LPP* and rs3130501 in *POU5F1* did differ between the Japanese and the Han Chinese populations (for rs6808574-C, 1.0 in Japanese vs. 0.985–1.0 in Han Chinese; and for rs3130501-G, 0.543 in Japanese vs. 0.662–0.680 in Han Chinese; 1000 genomes project data from http://www.1000genomes.org/; rs6808574 was excluded from the present Japanese study).

We did not observe any significant association of the six SNPs with susceptibility to type 2 diabetes in our Japanese population. Among these six SNPs, the effect direction of four of them (rs3130501 in *POU5F1*, rs4275659 near *MPHOSPH9*, rs6813195 near *TMEM154* and rs17106184 in *FAF1*) was consistent with that of the original report (OR > 1.0 adjusted for sex, age, and BMI; S4 Table), but rs9505118 in *SSR1* and rs702634 in *ARL15* showed inconsistent direction of effect with the prior study (OR < 1.0 adjusted for sex, age, and BMI; S4 Table).

The genotyping success rates for all SNPs were over 98.0% (S1 Table), and while the genotype concordance rates were 100% in duplicated samples (n = 64, see Materials and Methods), this discrepancy was not considered to be the consequence of technical error.

In our study, control individuals were younger than type 2 diabetes subjects; therefore our control group may have included individuals who will develop the disease later in life, which may increase the possibility of a type II error, although we included age as a co-variable in our logistic regression model. Then, we evaluated the association of the six SNPs with type 2 diabetes using older control individuals (age ≥ 40 years, ≥ 50 years, or ≥ 60 years). However, in these analyses, the effect sizes of the six SNP loci were almost the same as those in our original finding (S5 Table).We thereby deduced that our conclusions were not significantly skewed by the inclusion of the younger control individuals.

Because it has been shown that a genetic risk score (GRS) constructed from the sum of the number of risk alleles is a useful measure for evaluating effects of multiple candidate loci of interest in the underpowered sample [19, 20, 30], we constructed the genetic risk scores of our experimental subjects by summing up their number of risk alleles for the six SNPs. However, the association of GRS with type 2 diabetes was not statistically significant (p = 0.014, OR = 1.052, 95% CI 1.010–1.095; Table 2). The association of the six-loci GRS with type 2 diabetes is therefore probably not stronger than that of rs3130501 in *POU5F1* (p = 0.017) or rs4275659 near *MPHOSPH9* (p = 0.012) alone, suggesting that although the *POU5F1* and *MPHOSPH9* loci may individually have some effect on susceptibility to type 2 diabetes, the overall effect of the six SNP loci is not major in the Japanese.

The reasons for why the overall effect of the six SNP loci was not major in this Japanese population were not clear, but effect sizes of novel type 2 diabetes susceptibility loci in the transethnic GWAS were shown to be smaller (odds ratio = 1.06–1.10) in multiethnic populations [26] compared with those for type 2 diabetes loci identified in previous GWAS [3–12, 21–25]. Moreover, in the original transethnic GWAS meta-analysis, the Japanese sample size (n = 2,335) was much smaller than the European sample size (n = 69,033) [26], indicating the effect sizes in the original report might preferentially reflect effect sizes in European populations. Thus, the effects of these six SNP loci might be smaller among the Japanese, although heterogeneity in the effect sizes was not observed in the transethnic GWAS meta-analysis [26].

Estimated study power for this study to replicate the original association of each SNP with type 2 diabetes was between 23% and 58% when we set α = 0.05, and the prevalence of the disease was assumed to be 10% (S2 Table). Therefore, insufficient study power might explain the lack of statistically significant association of individual SNPs with the disease, especially for rs3130501, rs4275659, and rs6813195, whose effect sizes in this study were comparable to those in the original report (S4 Table); this could be a limitation of our present study.

In conclusion, we performed a replication study in a Japanese population for the association of six SNPs with type 2 diabetes, which were previously identified in a transethnic GWAS meta-analysis. Our results suggest that the six SNP loci derived from the transethnic GWAS meta-analysis do not have a significant effect on susceptibility to type 2 diabetes in the Japanese, although further evaluation for the association of the six loci using a larger Japanese population is required.

## Supporting Information

S1 Table Information of genotyping success rates for individual 6 SNPs. (DOCX)

S2 Table Power estimation for each SNP locus to replicate the results of original European study in the present study. Power estimation was performed using CaTS power calculator, CaTS: http://www.sph.umich.edu/csg/abecasis/CaTS/). The prevalence of type 2 diabetes is assumed to be 10%, α = 0.05. ^a^ Risk allele for type 2 diabetes reported in the original trans-ethnic GWAS. ^b^ Risk allele frequency in the Japanese population (controls) in the present study. ^c^ Information in the original trans-ethnic GWAS is shown.
(DOCX)

S3 Table Genotype distributions of 6 SNPs in case and control groups. HWE: Hardy-Weinberg equilibrium. ^a^ Risk alleles reported in the original trans-ethnic GWAS.
(DOCX)

S4 Table Association of 6 SNP loci with type 2 diabetes in a Japanese population and the original report. The results of logistic regression analysis are shown. ^a^ Risk allele reported in the original trans-ethnic GWAS. ^b^ Adjusted for age, sex and BMI. ^c^ Information in the original trans-ethnic GWAS is shown. RAF: Risk allele frequency.
(DOCX)

S5 Table Association study of 6 SNPs with type 2 diabetes using older control (age ≥ 40, n = 1,204, age ≥ 50, n = 906, age ≥ 60 n = 543). Results of logistic regression analysis using all type 2 diabetes participants (n = 5,817) are shown. ^a^ Risk allele reported in the original trans-ethnic GWAS. ^b^ Adjusted for age, sex and BMI.
(DOCX)
