# Supplementary material for: Replication Study in a Japanese Population of Six Susceptibility Loci for Type 2 Diabetes Originally Identified by a Transethnic Meta-Analysis of Genome-Wide Association Studies
Source: PLoS One. 2016 Apr 26;11(4):e0154093. doi: 10.1371/journal.pone.0154093 (PMC4845992; doi:10.1371/journal.pone.0154093)
Supplement: S1 Table — (DOCX) [file pone.0154093.s001.docx]

**Table S1.** Information of genotyping success rates for individual 6 SNPs.

| SNP | Nearby Gene | Success rate |
| --- | --- | --- |
| rs6813195 | *TMEM154* | 98.0% |
| rs9505118 | *SSR1* | 98.6% |
| rs17106184 | *FAF1* | 99.1% |
| rs3130501 | *POU5F1* | 98.1% |
| rs702634 | *ARL15* | 99.1% |
| rs4275659 | *MPHOSPH9* | 98.6% |
