# Supplementary material for: Replication Study in a Japanese Population of Six Susceptibility Loci for Type 2 Diabetes Originally Identified by a Transethnic Meta-Analysis of Genome-Wide Association Studies
Source: PLoS One. 2016 Apr 26;11(4):e0154093. doi: 10.1371/journal.pone.0154093 (PMC4845992; doi:10.1371/journal.pone.0154093)
Supplement: S2 Table — Power estimation was performed using CaTS power calculator, CaTS: http://www.sph.umich.edu/csg/abecasis/CaTS/). The prevalence of type 2 diabetes is assumed to be 10%, α = 0.05. a Risk allele for type 2 diabetes reported in the original trans-ethnic GWAS. b Risk allele frequency in the Japanese population (controls) in the present study. c Information in the original trans-ethnic GWAS is shown. (DOCX) [file pone.0154093.s002.docx]

**Table S2.** Power estimation for each SNP locus to replicate the results of original study

| SNP | Nearby Gene | Risk alleles ^a^ | RAF ^b^ | Reported OR ^c^ | Power |
| --- | --- | --- | --- | --- | --- |
| rs6813195 | *TMEM154* | C | 0.47 | 1.08 (1.06-1.10) | 58% |
| rs9505118 | *SSR1* | A | 0.56 | 1.06 (1.04-1.08) | 37% |
| rs17106184 | *FAF1* | G | 0.90 | 1.10 (1.07-1.14) | 32% |
| rs3130501 | *POU5F1* | G | 0.57 | 1.07 (1.04-1.09) | 47% |
| rs702634 | *ARL15* | A | 0.82 | 1.06 (1.04-1.09) | 23% |
| rs4275659 | *MPHOSPH9* | C | 0.67 | 1.06 (1.04-1.08) | 33% |

Power estimation was performed using CaTS power calculator, CaTS: http://www.sph.umich.edu/csg/abecasis/CaTS/)

The prevalence of type 2 diabetes is assumed to be 10%, α = 0.05

^a^ Risk allele for type 2 diabetes reported in the original trans-ethnic GWAS

^b^ Risk allele frequency in the Japanese population (controls) in the present study

^c^ Information in the original trans-ethnic GWAS is shown
