# Supplementary material for: Replication Study in a Japanese Population of Six Susceptibility Loci for Type 2 Diabetes Originally Identified by a Transethnic Meta-Analysis of Genome-Wide Association Studies
Source: PLoS One. 2016 Apr 26;11(4):e0154093. doi: 10.1371/journal.pone.0154093 (PMC4845992; doi:10.1371/journal.pone.0154093)
Supplement: S3 Table — HWE: Hardy-Weinberg equilibrium. a Risk alleles reported in the original trans-ethnic GWAS. (DOCX) [file pone.0154093.s003.docx]

**Table S3.** Genotype distributions of 6 SNPs in case and control groups

| SNP | Nearby gene | Allele1/Allele2  (Risk Allele ^a^) | Allele 11/12/22 | | P for HWE test | |
| --- | --- | --- | --- | --- | --- | --- |
|  |  |  | Type 2 diabetes | Controls | Type 2 diabetes | Controls |
| rs6813195 | *TMEM154* | A/G (G) | 1451/2851/1397 | 478/889/398 | 0.963 | 0.692 |
| rs9505118 | *SSR1* | C/T (T) | 1058/2793/1885 | 328/860/587 | 0.678 | 0.676 |
| rs17106184 | *FAF1* | C/T (C) | 4838/884/52 | 1476/293/11 | 0.102 | 0.388 |
| rs3130501 | *POU5F1* | A/G (G) | 909/2734/2065 | 305/885/580 | 0.935 | 0.298 |
| rs702634 | *ARL15* | A/G (A) | 3990/1594/182 | 1228/503/52 | 0.141 | 0.955 |
| rs4275659 | *MPHOSPH9* | G/A (G) | 2791/2448/497 | 801/806/171 | 0.224 | 0.121 |

HWE: Hardy-Weinberg equilibrium

^a^ Risk alleles reported in the original trans-ethnic GWAS
