# Supplementary material for: Replication Study in a Japanese Population of Six Susceptibility Loci for Type 2 Diabetes Originally Identified by a Transethnic Meta-Analysis of Genome-Wide Association Studies
Source: PLoS One. 2016 Apr 26;11(4):e0154093. doi: 10.1371/journal.pone.0154093 (PMC4845992; doi:10.1371/journal.pone.0154093)
Supplement: S4 Table — The results of logistic regression analysis are shown. a Risk allele reported in the original trans-ethnic GWAS. b Adjusted for age, sex and BMI. c Information in the original trans-ethnic GWAS is shown. RAF: Risk allele frequency. (DOCX) [file pone.0154093.s004.docx]

**Table S4.** Association of 6 SNP loci with type 2 diabetes in a Japanese population and the original report.

| SNP | Nearby  gene | Risk  Allele ^a^ | RAF | This study ^b^ | | Original report ^c^ | |
| --- | --- | --- | --- | --- | --- | --- | --- |
|  |  |  |  | *p* value | OR　　(95%CI) | *p* value | OR　(95%CI) |
| rs6813195 | *TMEM154* | C | 0.47 | 0.096 | 1.077 (0.987-1.174) | 4.1×10^-14^ | 1.08　　(1.06–1.10) |
| rs9505118 | *SSR1* | A | 0.56 | 0.513 | 0.971 (0.890-1.060) | 1.4×10^-9^ | 1.06　　(1.04–1.08) |
| rs17106184 | *FAF1* | G | 0.90 | 0.616 | 1.040 (0.893-1.210) | 4.1×10^-9^ | 1.10　　(1.07–1.14) |
| rs3130501 | *POU5F1* | G | 0.57 | 0.017 | 1.113 (1.019-1.215) | 4.2×10^-9^ | 1.07　　(1.04–1.09) |
| rs702634 | *ARL15* | A | 0.82 | 0.848 | 0.989 (0.883-1.108) | 6.9×10^-9^ | 1.06　 (1.04–1.09) |
| rs4275659 | *MPHOSPH9* | C | 0.67 | 0.012 | 1.127 (1.026-1.238) | 9.5×10^-9^ | 1.06　 (1.04–1.08) |

The results of logistic regression analysis are shown

^a^ Risk allele reported in the original trans-ethnic GWAS

^b^ Adjusted for age, sex and BMI

^c^ Information in the original trans-ethnic GWAS is shown

RAF: Risk allele frequency
