# Supplementary material for: Replication Study in a Japanese Population of Six Susceptibility Loci for Type 2 Diabetes Originally Identified by a Transethnic Meta-Analysis of Genome-Wide Association Studies
Source: PLoS One. 2016 Apr 26;11(4):e0154093. doi: 10.1371/journal.pone.0154093 (PMC4845992; doi:10.1371/journal.pone.0154093)
Supplement: S5 Table — Results of logistic regression analysis using all type 2 diabetes participants (n = 5,817) are shown. a Risk allele reported in the original trans-ethnic GWAS. b Adjusted for age, sex and BMI. (DOCX) [file pone.0154093.s005.docx]

**Table S5.** Association study of 6 SNPs with type 2 diabetes using older control (age ≥ 40, n=1,204, age ≥ 50, n=906, age ≥ 60 n=543)

| SNP | Nearby Gene | Risk Allele ^a^ | Control | *p* value ^b^ | OR (95%CI) |
| --- | --- | --- | --- | --- | --- |
| rs6813195 | *TMEM154* | C | age ≥ 40 | 0.125 | 1.075 (0.980-1.178) |
|  |  |  | age ≥ 50 | 0.077 | 1.096 (0.990-1.213) |
|  |  |  | age ≥ 60 | 0.480 | 1.047 (0.921-1.191) |
| rs9505118 | *SSR1* | A | age ≥ 40 | 0.400 | 0.961 (0.876-1.054) |
|  |  |  | age ≥ 50 | 0.314 | 0.949 (0.857-1.051) |
|  |  |  | age ≥ 60 | 0.225 | 0.923 (0.811-1.050) |
| rs17106184 | *FAF1* | G | age ≥ 40 | 0.777 | 1.023 (0.872-1.201) |
|  |  |  | age ≥ 50 | 0.505 | 1.061 (0.891-1.264) |
|  |  |  | age ≥ 60 | 0.410 | 1.097 (0.880-1.367) |
| rs3130501 | *POU5F1* | G | age ≥ 40 | 0.050 | 1.098 (1.000-1.204) |
|  |  |  | age ≥ 50 | 0.061 | 1.103 (0.995-1.222) |
|  |  |  | age ≥ 60 | 0.389 | 1.059 (0.929-1.208) |
| rs702634 | *ARL15* | A | age ≥ 40 | 0.675 | 0.974 (0.863-1.100) |
|  |  |  | age ≥ 50 | 0.682 | 0.972 (0.850-1.112) |
|  |  |  | age ≥ 60 | 0.714 | 0.968 (0.816-1.150) |
| rs4275659 | *MPHOSPH9* | C | age ≥ 40 | 0.016 | 1.129 (1.023-1.247) |
|  |  |  | age ≥ 50 | 0.026 | 1.132 (1.015-1.262) |
|  |  |  | age ≥ 60 | 0.433 | 1.057 (0.920-1.216) |

Results of logistic regression analysis using all type 2 diabetes participants (n=5,817) are shown

^a^ Risk allele reported in the original trans-ethnic GWAS

^b^ Adjusted for age, sex and BMI
